# Cell shape and maturation impacts α-actinin-2 tension in iPSC-derived cardiomyocytes

**DOI:** 10.1063/5.0304143

**Published:** 2026-02-11

**Authors:** Palash K. Dutta, Joshua M. Toth, Subramanian Sundaram, Xingyu Chen, Anant Chopra, Jourdan K. Ewoldt, Paige Cloonan, Xining Gao, Andrew S. LaCroix, Brenton D. Hoffman, Vivek B. Shenoy, Christine E. Seidman, Jonathan G. Seidman, Christopher S. Chen, Jeroen Eyckmans

**Affiliations:** 1 The Biological Design Center and Department of Biomedical Engineering, Boston University, Boston, Massachusetts 02215, USA; 2 The Wyss Institute for Biologically Inspired Engineering, Harvard University, Boston, Massachusetts 02215, USA; 3 Center for Engineering Mechanobiology and Department of Materials Science and Engineering, University of Pennsylvania, Philadelphia, Pennsylvania 19104, USA; 4 Department of Biomedical Engineering, Duke University, Durham, North Carolina 27705, USA; 5 Department of Cell Biology, Duke University, Durham, North Carolina 27710, USA; 6 Department of Genetics, Harvard Medical School, Boston, Massachusetts 02115, USA; 7 Division of Cardiovascular Medicine, Brigham and Women's Hospital, Boston, Massachusetts 02115, USA; 8 Howard Hughes Medical Institute, Chevy Chase, Maryland 20815, USA

## Abstract

The contractile activity of cardiomyocytes (CMs) critical to heart function emerges from the collective shortening of sarcomeres. However, how these sarcomeric forces are transmitted within CMs during this process remains poorly understood. Traction force microscopy has been used to measure overall forces exerted by CMs, but it falls short in providing insights into which specific proteins within sarcomeres transmit and whether cell shape influences forces within each sarcomere. Here, we aimed to characterize force generation on α-actinin-2, a z-disk protein that crosslinks anti-parallel actin filaments from adjacent sarcomeres and transmits force within a cell. By incorporating a Förster resonance energy transfer (FRET)-based molecular tension sensor in α-actinin-2, we measured contraction-induced deformation of the α-actinin-2 sensor in human-induced pluripotent stem cell-derived cardiomyocytes cultured on rectangular and circular adhesive patterns. We observed α-actinin-2 localized within sarcomeres, and actinin-2 loading correlated with sarcomere maturation and organization. α-actinin-2 tension increased in contracting rectangular-shaped cells, but not in circular cells. Moreover, the increase in tension was only observed in rectangular cardiomyocytes that were cultured for 5 days, and not after 24 h. Interestingly, the spread of FRET index values was increased in both rectangular and circular cells after 5 days in culture, compared to cells that were kept for 24 h in culture. Together, these data suggest that cell shape and maturation modulates tension on a load-bearing sarcomeric protein, α-actinin-2, and highlights the importance of characterizing tension across sarcomeric structures to understand cardiomyocyte contractile activity.

## INTRODUCTION

The collective shortening of sarcomeres drives cardiomyocyte contractility, which is essential for the heart's function to pump blood throughout the body.^1^ Sarcomeres—the smallest contractile units in muscle—are composed of myosin filaments tethered to myomesin (M-line) located at the center of the sarcomere and actin filaments anchored to α-actinin in the z-disk, which marks the boundaries of the sarcomere.^2,3^ The length of a sarcomere decreases as a result of the sliding of the actin filaments along the myosin filaments during contraction. In healthy cardiomyocytes, sarcomeres are longitudinally aligned into myofibrils, which are laterally registered at the Z-lines.^2,3^ This arrangement gives rise to the characteristic striated appearance of cardiomyocytes. Genetic mutations in sarcomeric proteins can result in impaired sarcomere alignment, which is referred to as sarcomeric disarray.^4–6^ Sarcomeric disarray is a hallmark of dilated and hypertrophic cardiomyopathies, two classes of cardiac diseases that have altered contractility.^4,5^ Similarly, in the event of hemodynamic overload, cardiac fibrosis, or myocardial infarction, maladaptive hypertrophy can drive sarcomeric disarray and compromised contractility.^5,7^ Therefore, the proper alignment of sarcomeres is a crucial factor in ensuring efficient contractility, and its disruption can lead to severe cardiac diseases and death.

In their native environment, cardiomyocytes exhibit a cylindrical morphology with a length-to-width ratio on the order of 7:1.^7^ At this ratio, cardiomyocytes show a uniaxial organization of myofibrils, as demonstrated in cardiomyocytes that have been seeded on micropatterned adhesive islands with different shapes.^8–10^ Conversely, cells with length-to-width ratios closer to one (e.g., square- or circular-shaped cells) exhibit a multiaxial myofibril arrangement. In fact, the organization of myofibrils in cardiomyocytes correlates well with the maximal principle stress directions that are dictated by the cell shape.^11^ Furthermore, traction force microscopy (TFM), a method to measure contractility that relies on deformation of elastic substrates on which cells are adhered,^12,13^ has revealed that cardiomyocytes with a 7:1 ratio exhibit maximum contractile forces, whereas contractility is reduced in cells with smaller or larger length-to-width ratios.^8^ Together, these studies have established the paradigm that cell shape regulates the contractility of cardiomyocytes by affecting myofibril alignment associated with maturation. However, it is unknown whether cell shape and myofibril alignment affect the magnitude of forces experienced by specific sarcomeric proteins within myofibrils, and whether molecular-scale loading reflects the macroscopy patterns of contractility observed at the cell level. Tools capable of reporting tension at the level of individual cytoskeletal components are therefore needed to begin addressing these questions.

TFM has played a crucial role in elucidating the correlation between the shape of cardiomyocytes, myofibril alignment, and contractility. However, conventional TFM techniques rely on substrate deformation, limiting its resolution, and thus have been unable to offer insights into force transmission inside subcellular structures. To overcome resolution limitations, genetically encoded Förster resonance energy transfer (FRET) tension sensors (TS) have been developed to measure tension across specific proteins of interest.^14^ FRET tension sensors consist of a donor and acceptor fluorophore that are covalently linked by a linker molecule. When the donor and acceptor are in close proximity (<10 nm), non-radiated energy transfer occurs from the donor to the acceptor, which yields high FRET efficiency.^15,16^ In contrast, when a force is applied to the linker, the linker extends, increasing the distance between the donor and acceptor, therefore reducing the FRET efficiency.^16,17^ The change in FRET efficiency is reflected by the ratio in fluorescent signal from donor vs acceptor, which, after careful calibration, correlates well with the force applied to the sensor.^14,18,19^ As such, FRET tension sensors have been effectively used to estimate the magnitude of forces across focal adhesion proteins such as vinculin,^18,20^ talin,^21^ and α-actinin,^22,23^ but the approach has not yet been used to measure tension experienced within sarcomeres.

In focal adhesions, α-actinins bind and stabilize actin to adhesion complexes and have been shown to play a crucial role in the transmission of mechanical forces from the cell to the extracellular matrix.^24^ Given that α-actinin-2 is responsible for cross-linking anti-parallel filamentous actin from adjacent sarcomeres at Z-discs in cardiomyocytes, we hypothesized that α-actinin-2 serves as a tension-bearing Z-disk protein, with its tension dependent on sarcomere alignment and maturation. To test this hypothesis, we developed a genetically encoded FRET tension sensor in α-actinin-2, expressed the construct in human-induced pluripotent stem cells (hiPSCs), and then differentiated the cells into cardiomyocytes. By using the sensor in conjunction with micropatterning and chemical cocktails to fix the cells in a contracted vs relaxed state, we demonstrate that α-actinin-2 loading correlates with sarcomere alignment and maturation of the cardiomyocytes. Finally, we employ a chemo-mechanical computational model to generate predictions of shape-dependent sarcomeric mechanics and find that they are consistent with the experimentally observed differences in α-actinin-2 loading and traction forces.

## RESULTS

### Design and characterization of α-actinin-2 tension sensor

**FIG. 1.**
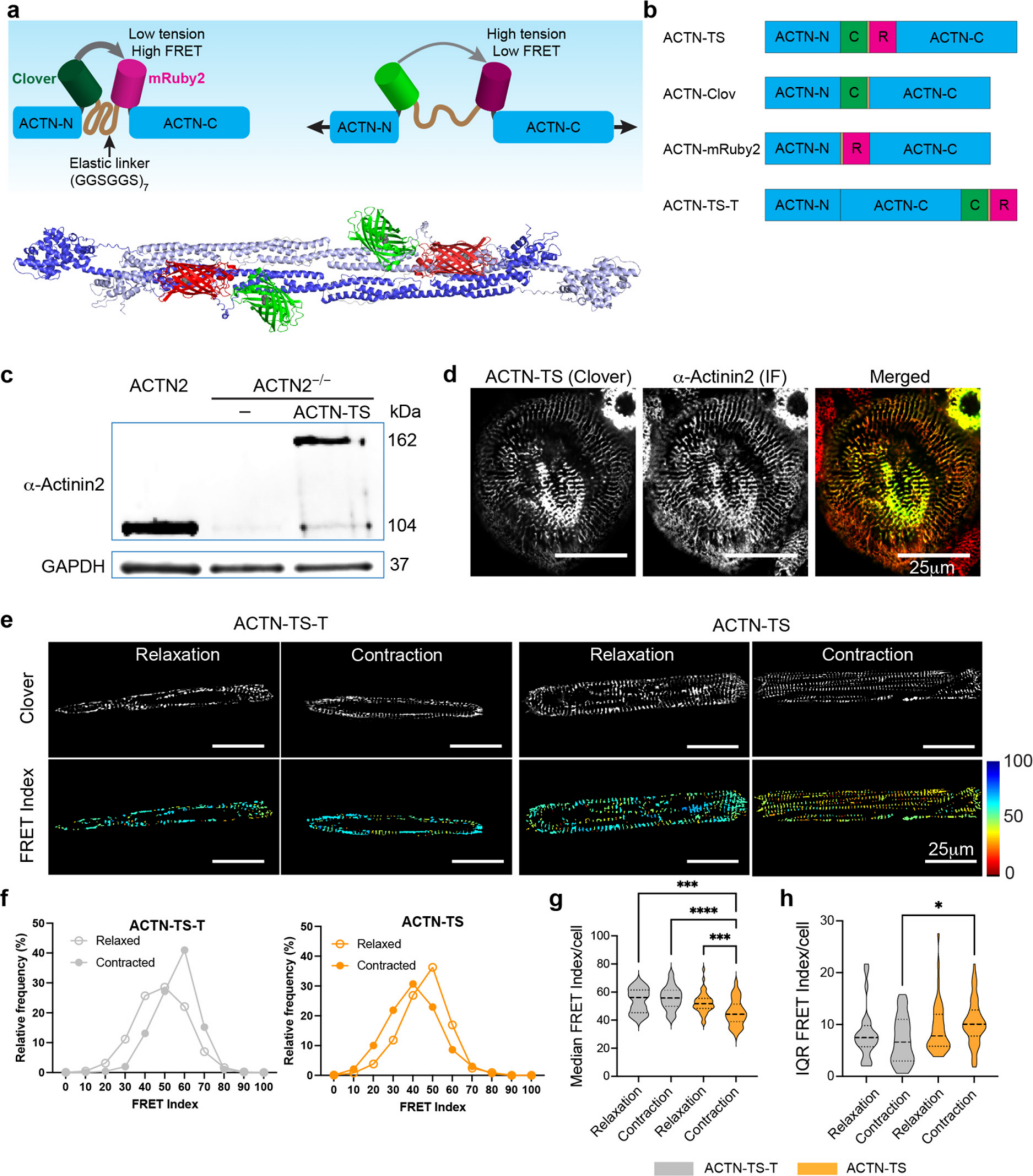
Construction and characterization of α-actinin-2 tension sensor (ACTN-TS). (a) The tension sensor (TS) consists of two fluorophores (Clover and mRuby2) separated by an elastic linker sequence (GGSGGS)_7_. The protein structure shows an α-actinin-2 dimer with a TS is inserted after amino acid 307 to create ACTN-TS (Protein Data Bank ID: 4D1E). (b) Schematics showing four constructs, ACTN-TS, α-actinin-2-Clover control (ACTN-Clov), α-actinin-2-mRuby2 control (ACTN-Ruby), and the TS inserted at the C-terminal of actinin (ACTN-TS-T) protein. (c) Western blot of lysates from CMs expressing ACTN2 (left), ACTN2^−/−^ CMs (middle), and ACTN2^−/−^ CMs expressing ACTN-TS. (d) Localization of signals from Clover in ACTN-TS and immunofluorescence of actinin antibody shows the correct formation of sarcomeres in hiPSC-derived cardiomyocytes (hiPSC-CM). (e) Fluorescent image of the clover showing sarcomeres in cells expressing ACTN-TS-T and ACTN-TS with corresponding FRET index images in contracted and relaxed states. (f) Frequency plots showing the FRET index of all sarcomeres in ACTN-TS-T control cells and ACTIN-TS cells in relaxed and contracted states. (g) Violin plots show the median FRET index/cell and interquartile range (IQR) of FRET index in rectangular hiPSC-CM in relaxed or contracted state. Kruskal–Wallis test. ^*^P < 0.05, ^**^P < 0.01, ^***^P < 0.001, ^****^P < 0.0001; 24 < n < 64 cells per condition.

α-actinin-2 is a 200 kDa dimer that crosslinks actin and titin filaments in the z-disk of sarcomeres. Each monomer comprises an N-terminal actin-binding domain, a central domain of four spectrin-like repeats, and a C-terminal calmodulin-like domain with two pairs of helix-loop-helix (EF) hand motifs. Due to its cylindrical appearance, the central domain is also known as the rod domain.^25^ To measure loading across α-actinin-2, we inserted a previously developed FRET force-sensitive biosensor,^18^ between arginine 307 (R307) and threonine 308 (T308) in the first spectrin-like repeat of the rod domain [Fig. 1(a)]. The actinin tension sensor (ACTN-TS) is composed of two fluorophores, Clover and mRuby2 that are separated by an elastic linker, (GGSGGS)_7_ [Fig. 1(a)]. In addition to the ACTN-TS, three control sensors were constructed [Fig. 1(b)]. The first two controls are ACTN-Clov (donor signal only) and ACTN-mRuby (acceptor signal only). The third control, ACTN-TS-T, has the TS attached to the C-terminal of α-actinin-2 rather than the rod domain; therefore, tension cannot be imparted to the TS.

To understand how the TS is integrated into α-actinin-2, we used AlphaFold3^26^ to model the protein structure of α-actinin-2 homodimer with the TS. Based on the amino acid sequence, AlphaFold3 predicted five protein structures [Fig. 1(a) and supplementary material Fig. 1]. For each model, the predicted structures for α-actinin-2 dimer, Clover, and mRuby2 were consistent with published crystal structures.^25,27^ The distance between the fluorochrome domains of the donor (V68 of Clover) and the acceptor (A62 of mRuby2) in the same α-actinin-2 monomer varied from 3.12 to 4.98 nm, which is within the Förster radius of 6.32 nm (the distance at which there is 50% energy transfer). However, the distances between donor and acceptor fluorophores on different homomers ranged from 7.21 to 12.56 nm [supplementary material Fig. 1(b)]. The variability in the distances between the chromophores likely stems from the flexible (GGSGGS)_7_ linker domain, which allows for translation and rotation of the fluorophores relative to each other. This variability may raise a concern that intermolecular FRET could emerge between donor and acceptor from different FRET pairs when the distance between the donor and acceptor is below 10 nm. However, studies using a similar FRET sensor inserted at aa 300 in α-actinin-1 showed no evidence of intermolecular FRET.^28^ Because we cannot rule out potential interference of intermolecular FRET, we report relative FRET index values instead of absolute FRET efficiency values.

To determine whether the exogenously expressed ACTN-TS affects sarcomerogenesis, we employed CRISPR/Cas9 gene editing to generate hiPSC-derived cardiomyocytes that lacked α-actinin-2. Knockdown of endogenous α-actinin-2 in this cell line was confirmed with Western blot [Fig. 1(c)] and immunofluorescent staining (supplementary material Fig. 2). In contrast, lysate from cells expressing ACTN-TS in these knockout cells showed a strong band at 162 kDa. The reduced mobility compared to WT control cells (105 kDa) was due to the incorporation of the TS into α-actinin-2. Immunofluorescent staining for actinin showed that the ACTN-TS was properly recruited to the Z-disk, as the signal from Clover in the TS was indistinguishable from the immunofluorescent signal from antibody-stained α-actinin-2 [Fig. 1(d)]. Furthermore, the cell morphology and myofibril organization were similar in cells expressing ACTN-TS vs wild-type cells expressing endogenous α-actinin-2 (supplementary material Fig. 2). Together, these data indicate that the ACTN-TS did not impair sarcomere assembly.

**FIG. 2.**
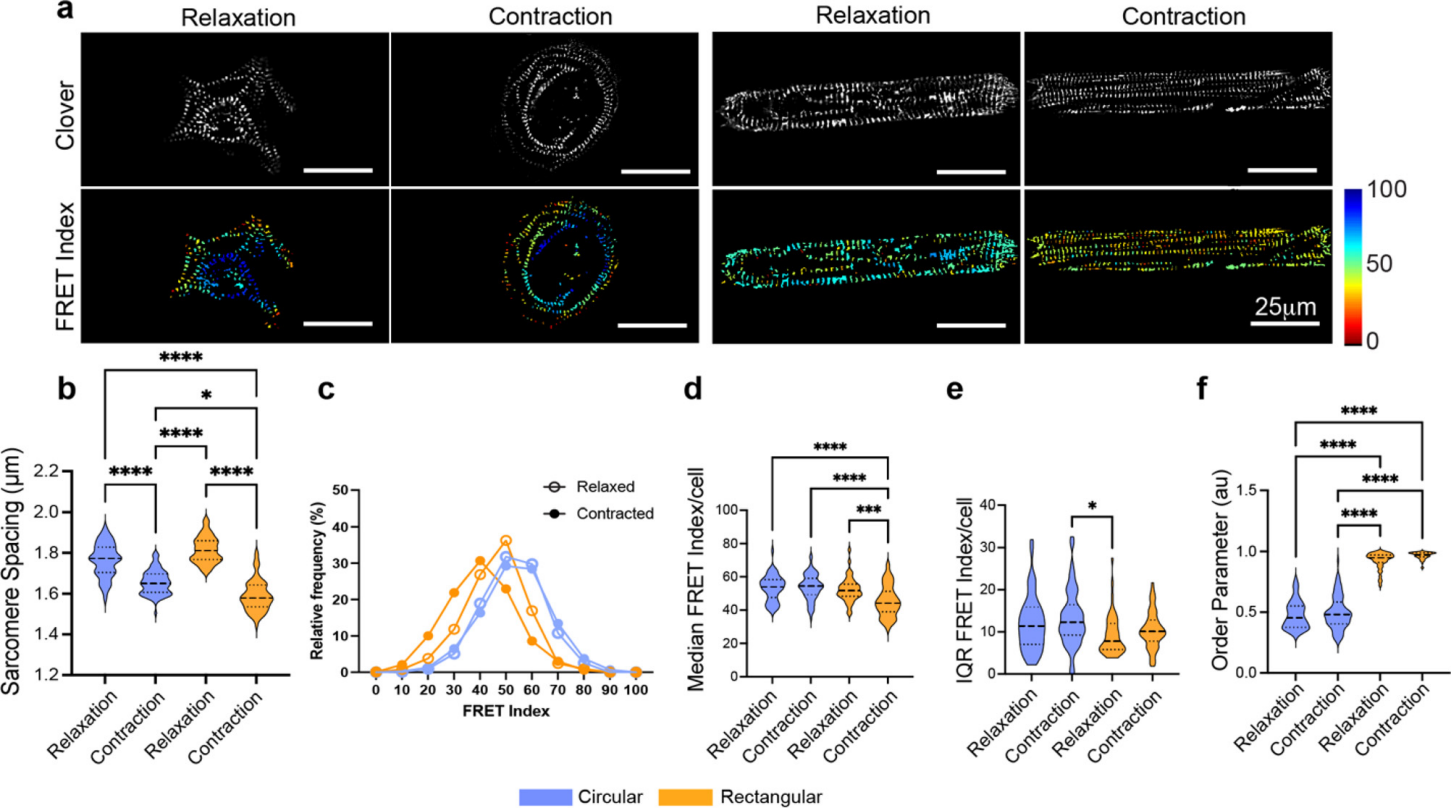
Sarcomeric organization and tension in shape-patterned CMs. (a) Fluorescent images (clover) and FRET index of circular and rectangular patterned hiPSC-CMs in relaxation and contraction state. (b) Violin plots of sarcomere spacing, (c) distribution plot of FRET index, (d) violin plots of median FRET index/cell, (e) IQR, and (f) order parameter (arbitrary units) in circular (blue) and rectangular (orange) shaped hiPSC-CMs in relaxation and contracted state. ^*^P < 0.05, ^**^P < 0.01, ^***^P < 0.001, ^****^P < 0.0001, by Kruskal–Wallis test; 32 < n < 78 cells per condition.

Next, we sought to investigate whether the ACTN-TS sensor could be used in cardiomyocytes that were fixed in a relaxed vs a contracted state as measuring FRET in beating cardiomyocytes was technically challenging. Briefly, hiPSC-derived cardiomyocytes that harbored either ACTN-TS or the non-sensing control ACTN-TS-T were seeded onto rectangular ECM patterns to consistently measure fluorescent signal without confounding cell shape effects. After 5 days in culture, cells were fixed in a contracted state using 20 mM caffeine and 1.5% paraformaldehyde in Tyrode's buffer, or in a relaxed state using 50 mM KCl for 1 h followed by fixing with 1% PFA. To quantify the extent of contraction upon addition of the contraction cocktail, sarcomere spacing was measured and compared to that of live cells. Sarcomere spacings in live beating cells [contracted:1.63 ± 0.15 *μ*m (mean ± SD); relaxed: 1.73 ± 0.14 *μ*m] and fixed cells (contracted: 1.61 ± 0.15 *μ*m; relaxed: 1.74 ± 0.24 *μ*m) showed no significant differences (supplementary material Fig. 3), validating the use of fixed cells for FRET analysis.

**FIG. 3.**
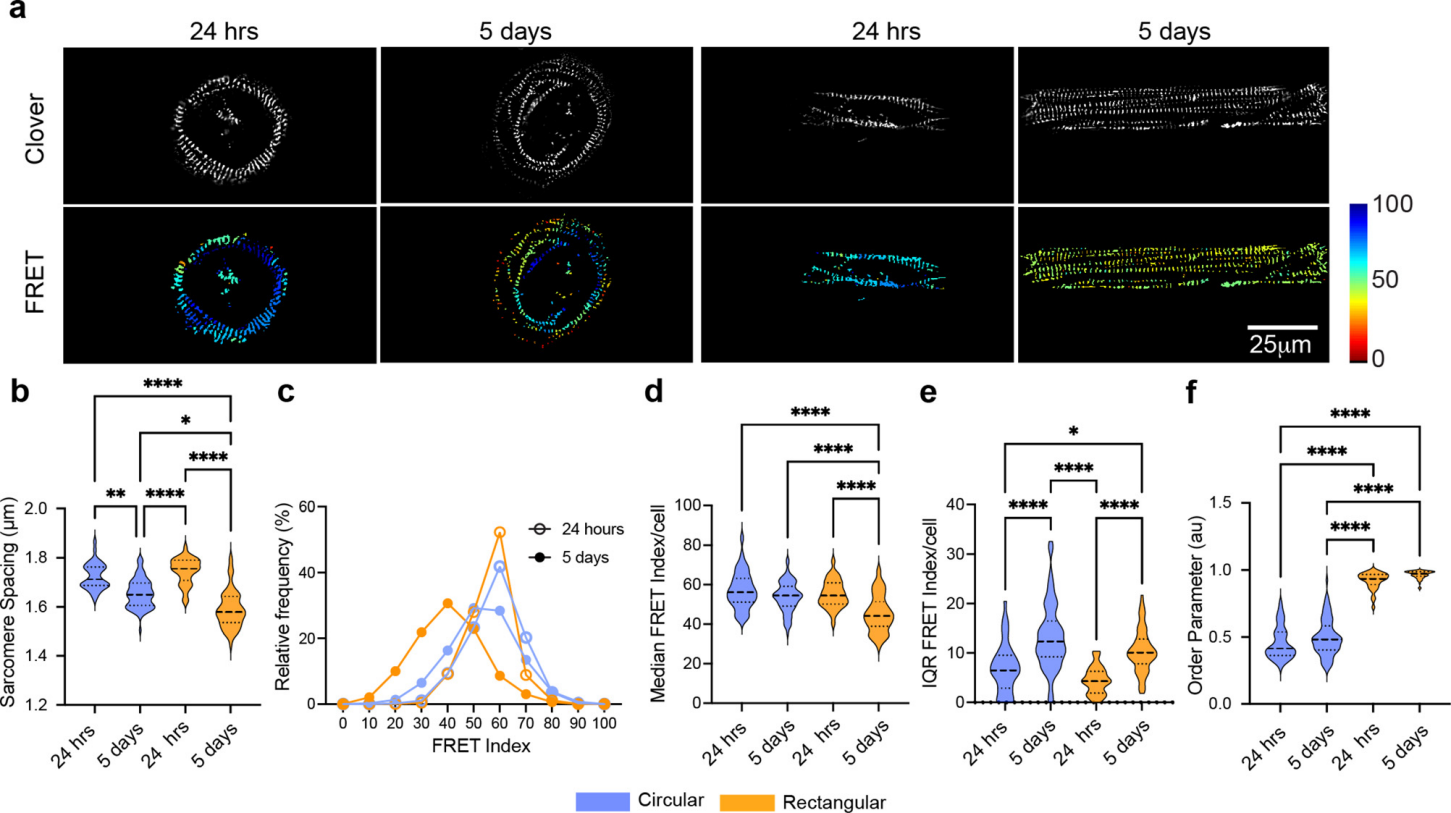
FRET analysis of sarcomeres in immature and mature iPSC cardiomyocytes. (a) Fluorescent images (clover) and FRET index of circular and rectangular patterned hiPSC-CMs in contracted state 24 h and 5 days after plating. (b) Violin plots of sarcomere spacing, (c) distribution plot of FRET index, (d) violin plots of median FRET index/cell, (e) IQR of FRET index/cell, and (f) order parameter (arbitrary units). ^*^P < 0.05, ^**^P < 0.01, ^***^P < 0.001, ^****^P < 0.0001 by Kruskal–Wallis test. 32 < n < 78 cells per condition.

Using this approach, we measured FRET in hiPSC cardiomyocytes incorporated with ACTN-TS-T vs ACTN-TS to assess the efficacy of the tension sensor [Figs. 1(e) and 1(g)]. For each cell, we report both the median FRET index and the interquartile range (IQR) of FRET index values. Whereas the median reflects the typical α-actinin-2 loading per cell, the IQR quantifies the dispersion of FRET values among sarcomeres within that cell and thus provides a measure of intracellular heterogeneity in Z-disk loading. In cells with ACTN-TS-T, the distribution of the FRET index values shifted from normal in relaxed cells to skewed in contracted cells [Fig. 1(f)], without affecting the median and IQR FRET index per cell [Figs. 1(g) and 1(h)]. In contrast, hiPSC cardiomyocytes harboring ACTN-TS showed a significant reduction in FRET index in the contracted state (44.21, 95% CI [43.07–47.75]) compared to the relaxed state (51.70, 95% CI [48.36–55.55]), suggesting that α-actinin-2 was under tension in contracted cardiomyocytes [Figs. 1(f) and 1(g)]. The dispersion of the FRET index values as quantified by the IQR was significantly increased in contracted cardiomyocytes harboring the ACTN-TS compared to cells with the control sensor [Fig. 1(h)]. These findings confirm ACTN-TS as a valid tension sensor to study tension on actinin-2 in sarcomeres.

### FRET analysis in rectangular and circular cardiomyocytes

Having established that α-actinin-2 experiences tension during sarcomere contraction, we next asked how cell shapes affect α-actinin-2 tension since cell shape regulates sarcomere alignment and cardiomyocyte contractility.^8,29^ To address this question, hiPSC cardiomyocytes expressing ACTN-TS were cultured on rectangular and circular extracellular matrix (ECM) patterns for 5 days, and sarcomere alignment and FRET were assessed in relaxed and contracted states after fixation [Fig. 2(a)]. In agreement with previous studies, sarcomeres in rectangular cardiomyocytes showed a higher degree of alignment, compared to circular cardiomyocytes, as indicated by a higher order parameter [Fig. 2(f)]. The order parameter was not affected by the contractile state of the cells. While sarcomere spacing confirmed contraction with shorter spacing in contracted vs relaxed cardiomyocytes for both cell shapes [Fig. 2(b)], rectangular cardiomyocytes exhibited shorter sarcomere spacing than circular cells, suggesting that rectangular cells shorten sarcomeres more efficiently than circular cells. Interestingly, despite differences in sarcomere shortening, circular cells showed no significant difference in the median FRET index between contracted (0.9795, 95% CI [0.9488–1.005]) and relaxed states (0.9771, 95% CI [0.9352–1.042]). In contrast, rectangular cardiomyocytes exhibited a significantly lower FRET index in the contracted state (0.8339, 95% CI [0.7764–0.8649]) compared to the relaxed state (0.9494, 95% CI [0.9068–0.9702]). The IQR of the FRET index values was comparable, suggesting that neither cell shape nor contraction state affected the dispersion of FRET index [Fig. 2(d)]. These results suggest that the ACTN-TS experiences tension during contraction in rectangular cardiomyocytes but not in circular cells, indicating that α-actinin-2 is a load-bearing protein in the Z-disk.

### Tension experienced by α-actinin-2 in immature and mature sarcomeres in hiPSC-CM

All experiments so far were conducted 5 days after plating because myofibrils in human-induced pluripotent stem cell-derived cardiomyocytes (hiPSC-CMs) are fully established and mature at this timepoint.^30^ However, since α-actinin-2 is a critical component for the assembly of sarcomeres emanating from costameres during early sarcomerogenesis,^30^ we investigated whether cell shape affects the tension on α-actinin-2 in cardiomyocytes with less mature sarcomeres. To address this question, we measured sarcomere spacing, FRET index, and order parameter in contracted hiPSC cardiomyocytes 24 h or 5 days after plating on circular and rectangular ECM patterns. Both 1-day and 5-day hiPSC cardiomyocytes contracted after fixation with our contraction cocktail, but 5-day cells had shorter sarcomere spacing than 1-day cells [Figs. 3(a) and 3(b)]. While there was no significant change in sarcomere spacings between rectangular and circular cells after 24 h, there was a reduction in sarcomere spacing and a decrease in FRET index only in rectangular cells after 5 days in culture [Figs. 3(c) and 3(d)]. Interestingly, the IQR increased over time for sarcomeres in both circular and rectangular cells, but the degree of sarcomere alignment was not different between 1-day and 5-day cardiomyocytes [Figs. 3(d) and 3(e)]. Collectively, these data suggest that cell shape controls the early sarcomere organization, while tension on α-actinin-2 is only imparted in mature cardiomyocytes.

### Modeling shape-dependent relationships between α-actinin-2 tension and whole-cell contractility

**FIG. 4.**
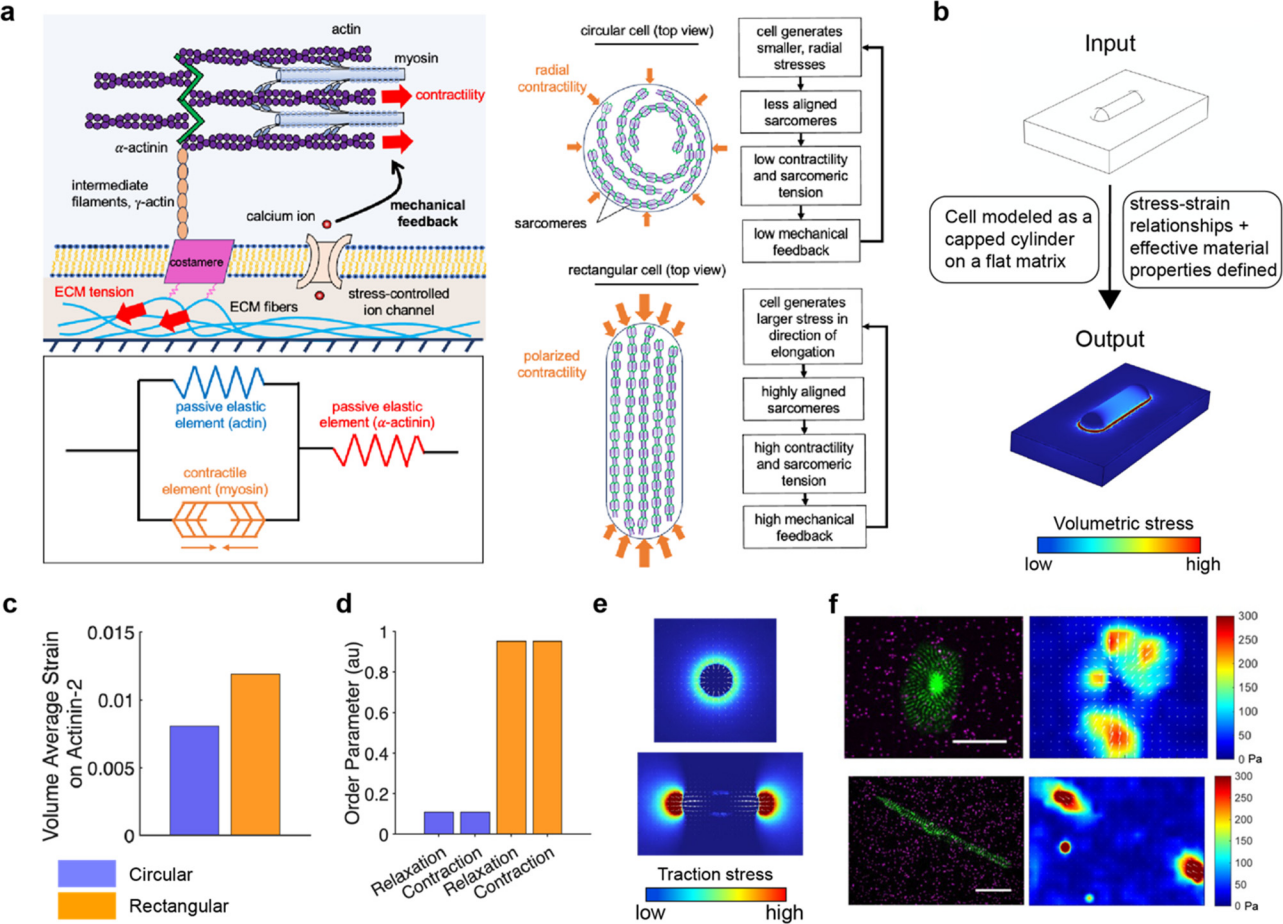
Simulations predict sarcomere tension and cell-generated substrate tractions. (a) The chemo-mechanical feedback model for cellular contractility. Adherent cells generate stress against their external environment that activates mechanosensitive pathways such as stretch activated calcium channels, creating positive feedback between myosin activation and external stress. Mechanically, the sarcomere is modeled as a contractile myosin element in parallel with an elastic actin element and in series with an elastic α-actinin element as shown. (b) Implementation of the chemo-mechanical feedback model for cellular contractility within a coarse-grain finite element framework. The system is solved for isotropic bulk and shear moduli to be applied to the geometry of the cell in COMSOL, which consists of half of a spherically capped cylinder resting on a block of substrate. (c) Predicted volume-averaged total volumetric strain on α-actinin-2 in circular and rectangular cells. (d) Predicted order parameter in contracted and relaxed circular and rectangular cells. (e) Simulated traction stresses for rectangular and circular cells measured near the substrate surface. White arrows indicate the predicted substrate displacement field. (f) Images of circular and rectangular cells on fluorescent microbeads (magenta) along with heat maps of substrate traction (Pa) during cell contraction that are estimated with traction force microscopy (scale bar, 25 *μ*m).

After establishing the impact of cell shape and cardiomyocyte maturation on α-actinin-2 tension, we next asked how these experimentally observed differences relate to the stresses generated at the whole-cell level. To explore this question, we employed a computational mechanics model, i.e., a modified version of the chemo-mechanical feedback model originally developed by Shenoy *et al.*^31^ for non-muscle myosin II, to simulate cardiomyocyte contractility and examine whether predicted variations in sarcomeric strain and alignment are consistent with the trends observed in our FRET and TFM measurements. This model describes how contractility, strain, and free energy evolve when a cell adheres to a substrate and applies contractile forces. Briefly, it establishes a positive feedback loop between non-muscle myosin motor activation and external stresses at the cell–matrix interface, mediated through mechanosensitive signaling pathways^32–35^ [Fig. 4(a)]. This feedback loop is energetically feasible due to the coupling of motor recruitment to ATP consumption, prompting the cell to favor a more contractile state and a higher equilibrium density of active motors to lower its free energy.^36,37^

While this model abstracts Rho/ROCK and calmodulin signaling as the chemical activators of myosin, which is well supported for non-muscle myosin II, cardiac myosin is primarily controlled by calcium-mediated thin filament activation through troponin. However, chemo-mechanical feedback loops where external mechanical cues can modulate biochemical activation of myosin remain highly relevant in the cardiac context as well. For instance, mechanical stress in cardiomyocytes can activate intracellular signaling, including stretches affecting calcium influx, ultimately modulating actomyosin interactions. Thus, while the specific molecular modulators differ in prominence between non-muscle and cardiac muscle settings, the broader paradigm where cell contractility is shaped by feedback between mechanical load and biochemical myosin activation remains applicable, and thus, we employed the model accordingly.

In our adaptation, the free energy of the cell-matrix system is considered as the sum of the strain energy of the sarcomeres and matrix, the chemical energy required for myosin to bind to actin, the work performed by motors, and the energy released when motors hydrolyze adenosine triphosphate (ATP) [Eq. (1) in Methods]. Employing tensorial forms for cytoskeletal stress, $\sigma_{ij}$, strain, $\epsilon_{ij}$, and contractility, $\rho_{ij}$, enables our 3D implementation to study cell-shape-dependent mechanical behavior and stress distribution.

To evaluate the strain on sarcomeric components, we employ a three-element model reflecting the mechanical arrangement of actin, myosin, and α-actinin. Contractile elements representing myosin motors are modeled as force dipoles in parallel with passive elastic elements (actin filaments) and in series with other passive elastic crosslinkers such as α-actinin-2 [Fig. 4(a)]. It is important to note, however, that the sarcomere encompasses a range of actin–myosin cross-linking proteins beyond α-actinin-2, including proteins like titin and other Z-disk components. Therefore, our FRET-based measurements with α-actinin-2 only partially capture the total tension experienced by the sarcomere during contraction and should be interpreted as a representative, but incomplete, reflection of the sarcomeric tension distribution.

By minimizing the total chemo-mechanical free energy, we derived steady-state stress–strain relationships and material properties from the chemo-mechanical model, which we then used as input for a coarse-grained finite element model to calculate the stress and strain fields generated by cardiomyocytes of different shapes [Fig. 4(b)]. Our simulations predict that the total volumetric average strain on α-actinin-2 is greater in contracted rectangular cells than in contracted circular cells [Fig. 4(c)]. Assuming that sarcomeres predominantly align along the directions of principal stress within the cell body,^11^ our modeling approach allowed us to calculate an order parameter as a proxy for sarcomere alignment. Simulations yield a substantially larger order parameter for rectangular than circular cells in both the contracted or relaxed states [Fig. 4(d)], confirming experimental observations [Fig. 2(e)]. Furthermore, predicted substrate traction forces show that rectangular cells generate high tractions at their longitudinal ends, while circular cells produce lower, more isotropically distributed forces [Fig. 4(e)]. Our experimental TFM measurements in cells patterned on rectangular or circular substrates closely match these computational predictions [Fig. 4(f)].

By validating the computational model against experimental data, we demonstrate the utility of a chemo-mechanical feedback modeling approach, even one adapted from a non-muscle myosin context, in predicting key biomechanical features of cardiomyocytes. This model framework captures how cell geometry modulates tension and traction at the sarcomeric and cellular levels, despite underlying differences in the regulatory details of myosin activation and the molecular specificity of the FRET marker used.

## DISCUSSION

The results of this study indicate that cell shape and the maturation of sarcomeres have a large impact on sarcomere length, alignment, and load on α-actinin-2. Rectangular cells with a 7:1 aspect ratio demonstrate a significant improvement in the sarcomeric organization and order, increased shortening of sarcomeres, and elevated tension on α-actinin-2 over time, all consistent with improved contractility. In contrast, although circular cells exhibit shorter sarcomere spacing in the contractile state, they do not show substantial improvements in sarcomeric order or changes in tension on α-actinin-2 over time, suggesting a relative lack of maturation.

These findings suggest that the organization of sarcomeres in rectangular cells allows for their maturation and stabilization, which does not occur in circular cells. Previous work has shown that forces are critical for sarcomerogenesis and stabilization of sarcomeres, as disruption of β-cardiac myosin-mediated force generation in maturing hiPSC cardiomyocytes results in disassembly of sarcomeres.^30^ Thus, it is possible that circular cells do not generate sufficient force or tension to sustain sarcomere stabilization. Predictions of order parameter extracted from our simulations further support this hypothesis, as the order parameter calculated purely from predicted principal stress directions in the cell shows good agreement with the experimentally measured order parameter, supporting the hypothesis that sarcomeres tend to align along the directions of principal stresses in the cell body. Therefore, it is tempting to speculate that the inability of circular cells to stabilize could be attributed to a deficiency in a mechanical feedback loop that regulates sarcomere stabilization through, for example, unfolding of sarcomeric scaffolding proteins.

Although TFM has been extensively employed for measuring cardiomyocyte contractility, this work illustrates how the use of FRET sensors provides new insights into the distribution of loads at a molecular level within sarcomeres. Here, we observed changes in loading on α-actinin-2 within the Z-discs of sarcomeres. Intriguingly, whereas we observed a reduction in sarcomere spacing in contraction compared to relaxation, or in the contracted state 24 h after plating for both cell shapes, the loading and deformation of α-actinin-2 occurred only in contracting mature rectangular cells and not in circular cells. These findings suggest that α-actinin-2 may experience tension and unfold only at a higher threshold of force than required for sarcomere shortening. Given that sarcomeres are composed of 478 proteins,^38^ we speculate that several structural sarcomeric proteins, such as titin,^38^ may experience loading and deformation before α-actinin-2 does. FRET tension sensors, such as the one presented herein, could be used to begin to catalog tension-bearing sarcomere proteins to gain insights into how mechanical forces are distributed among sarcomeric structures during contraction.

As with any reductionist approach, our study has several limitations that also point toward future opportunities. Our primary goal was to develop and validate a genetically encoded α-actinin-2 tension sensor in hiPSC cardiomyocytes and to examine how controlled geometric constraints modulate sarcomeric loading. For this initial validation, we intentionally selected two geometries, 7:1 rectangles and circles, that represent opposite ends of the spectrum of cellular anisotropy. Rectangles approximate the physiological aspect ratio of adult cardiomyocytes and promote aligned myofibrils, anisotropic force transmission, and maximum contraction, whereas circles provide a non-physiological, isotropic condition that disrupts sarcomeric alignment.^8^ While these two shapes offer a well-controlled framework for probing how geometric extremes influence sarcomere maturation and Z-disk loading, they do not capture the broader diversity of physiological and pathological cardiomyocyte morphologies. In addition, FRET imaging was performed on rigid PDMS substrates to ensure stable micropatterning and reproducible quantitative imaging; however, native cardiomyocytes reside within compliant and dynamically deforming environments, and substrate mechanics are known to influence maturation and contractile behavior. Similarly, this study examined only wild-type hiPSC cardiomyocytes, and future work incorporating disease-associated mutations on deformable substrates will be important for determining how pathological remodeling alters sarcomeric loading.

In conclusion, our work suggests that cell shape directly affects sarcomeric force generation in addition to sarcomere alignment,^8^ which impacts the cardiomyocytes' overall contractility. Moreover, this study highlights the use of FRET sensors to explore the intricate relationships between sarcomere organization, force transmission within the sarcomere, and cardiomyocyte maturation. Together, these findings underscore the complex interplay between cellular structure and function in cardiomyocytes, and the use of FRET sensors offers a promising avenue for further investigating the underlying mechanisms driving sarcomere dynamics and cardiomyocyte maturation.

## METHODS

### Construction of tension sensors

Second-generation viral packaging plasmids psPAX2 (Addgene plasmid #12260) and pMD2.G (Addgene plasmid #12259) were purchased from Addgene. The TS module^18^ and α-Actinin-2 (ACTN2-pEGFP, Addgene plasmid #52669) fragments were generated via polymerase chain reaction (PCR). The primers to generate the fragments are shown in the supplementary material Table 1. The plasmid fragments were purified and incorporated into pRRL lenti-backbone^18^ using Gibson Assembly. The assembled DNA fragments were transformed into *E. coli* cells (One Shot TOP10-*E. coli*, Cat No. C4040-10) and was verified for single colonies by DNA sequencing. The ACTN-TS or ACTN-TS-T or ACTN-Clov or ACTN-Ruby or ACTN-KO, psPAX2, and pMD2.G plasmids were co-transfected into HEK293-T cells using calcium phosphate transfection. After 6 h, the transfection mixture was exchanged for full media. After an additional 48 h, media containing lenti-viral particles were harvested using PEG-it (SBI, # LV810A-1) precipitation, resuspended in cold PBS, and stored at −80 °C.

**TABLE I.** Computational model parameters.

Parameter	Description	Value
α	Strength of mechanosensitive signaling coupled to non-muscle actomyosin contractility	$1\;{\text{kPa}}^{-1}$
$\alpha^{a}$	Strength of mechanosensitive signaling coupled to sarcomere contractility	$2\text{ kP}a^{-1}$
$\rho_{0}$	Quiescent state contractility	2 kPa
K	Cytoskeleton bulk modulus	3.333 kPa
μ	Cytoskeleton shear modulus	0.3448 kPa

### Photopatterning

Micropatterned substrates were prepared as described previously.^8,39^ Briefly, polydimethylsiloxane (PDMS) stamps were created from patterned silicon masters that were generated using photolithography. Glass substrates were spin coated with PDMS and polymerized in a 60 °C oven overnight. PDMS stamps were coated with 50 mg/ml fibronectin for 1 h at room temperature and stamped onto PDMS-coated glass substrates that were pre-activated in a UV-Ozone cleaner (Jelight) for 7 min. Patterned substrates were treated with 0.2% pluronic F127 (Sigma) for 1 h before cell seeding to prevent nonspecific adhesion of cells to the un-patterned areas of the substrate.

### Cell culture, lentiviral transduction, and fixation

Cardiomyocyte cell differentiation and culture: PGP1 hiPSCs (Coriell Institute GM23338) were maintained in mTESR-1 (StemCell) on Matrigel-coated tissue culture plates and were differentiated into CMs as described previously.^40^ Specifically, iPSCs were differentiated in Roswell Park Memorial Institute (RPMI) 1640 medium (Thermo Fisher Scientific) supplemented with B27 minus insulin (Thermo Fisher Scientific) by sequential targeting of the WNT pathway—activating the WNT pathway using 12 *μ*M of CHIR99021 (Tocris) on Day 0 for 24 h and inhibiting the WNT pathway using 5 *μ*M of IWP4 (Tocris) on Day 3 for 48 h. Insulin was added on Day 7. CMs were isolated after showing spontaneous beating (between Day 9 and Day 14) using metabolic selection by adding 4 mM of dl-lactate (Sigma-Aldrich) in glucose-free RPMI 1640 medium (Thermo Fisher Scientific) for 4 days. Following selection, CMs were passaged onto six-well plates coated with fibronectin (10 *μ*g/ml; Corning), maintained in RPMI 1640 medium supplemented with B27 (Thermo Fisher Scientific), and used for assays 2 weeks after passaging around Day 30 after initiation of differentiation.

To delete WT actinin specifically and not actinin 2 provided by the TS constructs, iPSCs were transduced with ACTN-KO lentiviral construct containing CRISPR-cas (lentiCRISPRv2 system, Addgene plasmid #52961) and a guide RNA (gRNA: 5′-GTACAACTACGTGTACGACG-3′). CMs were transduced in growth medium overnight, and medium was replaced the following morning. Four days post-transduction, cells were treated with 2 *μ*g/ml puromycin for another 4 days to select for transduced cells.

To fix beating CMs in a contracted state, RPMI medium from the culture well was replaced with Tyrode's buffer (37 °C). After 30 min of incubation, half of the volume of Tyrode's buffer was replaced with Tyrode's buffer (37 °C) containing 40 mM caffeine and 3% paraformaldehyde (PFA; final concentration: caffeine 20 mM and PFA 1.5%). Wells were washed with PBS after 5 min. Similarly, to fix CMs in a relaxed state, the culture medium was replaced with Tyrode's buffer with 50 mM K^+^ (relaxation buffer) at 37 °C for 30 min. After incubation, half of the volume of relaxation buffer was replaced with 2% PFA in relaxation buffer (37 °C) to make a final concentration 1%. Samples were washed in phosphate buffered saline (PBS) after 5 min of fixation.

### Immunofluorescence

Cardiomyocytes seeded on fibronectin-coated glass surface were fixed with 4% PFA, followed by permeabilized in 0.1% Triton-X in PBS for 5 min at room temperature. The cells were rinsed in PBS and blocked for 1 h at room temperature in 2% bovine serum albumin (BSA) in PBS. Primary α-actinin-2 rabbit antibody (ThermoFisher #701914) at a 1:500 dilution in 2% BSA in PBS was applied for 12 h at 4 °C. The cells were rinsed in PBS. Secondary antibody (goat anti-rabbit IgG antibody Alexa Fluor 647, Life Technologies) at a 1:1000 dilution in 2% BSA in PBS was applied for 2 h at room temperature and then rinsed three times in PBS and left in PBS for imaging.

### Western blot

Western blotting for α-actinin was conducted following standard protocols. Briefly, cells were washed twice with cold PBS in a 12-well plate prior to lysis with 60 *μ*l of lysate buffer, consisting of IP lysis buffer and a 1/100 dilution of protease/phosphatase inhibitor cocktail (#5872, Cell Signaling). An additional 60 *μ*l of lysate buffer was added to each well, and the resulting mixture was collected in the same tube. This 2 × 60 μl mix was prepared for each sample and kept at 4 °C for 30 min with agitation. Following this, the samples were centrifuged at 14 000 g for 10 min. The supernatant was carefully collected into a new tube, and the tubes were frozen using dry ice. The samples were composed of 43.2 *μ*l of the collected mixture, 1.8 *μ*l of 2-mercaptoethanol, and 15 *μ*l of NuPAGE Lithium Dodecyl Sulfate (LDS) buffer (4×). The mixture was thoroughly mixed and heated at 95 °C for 8 min and then cooled down to room temperature. A 20 *μ*l aliquot of the mixture was loaded into a well in a 1.5 mm gel, along with 5 *μ*l of Novex Sharp pre-stained protein standard. Transfer was performed using the BioRad TransBlot Turbo system. The membrane was initially washed with tris buffered saline with tween-20 (TBST) buffer and then incubated with TBST–5% Milk–2.5% BSA for 1 h. The alpha Actinin 2 Recombinant Rabbit Monoclonal Antibody (7H1L69) was diluted 500-fold in TBST-5% BSA buffer (stock concentration: 0.5 mg/ml, 10 ml). The TBST–Milk–BSA buffer was replaced with the buffer containing the primary antibody without washing the membrane, which was then left overnight at 4 °C. The membrane was subsequently washed three times with 15 ml of TBST for 10 min each. The secondary antibody, anti-rabbit IgG-HRP-linked antibody, was diluted 1000-fold in TBST–5% Milk. The membrane was incubated in the secondary antibody solution for 1.5 h at room temperature. After the incubation, the membrane was washed three times for 10 min each with TBST. A mixture of 3 ml of luminol solution and peroxide solution (SuperSignal West Pico PLUS Chemiluminescent substrate, ThermoFisher) in a 1:1 ratio was poured onto the membrane, and after 4 min, the solution was discarded. The membrane was imaged, and subsequent washing with TBST was performed for 2–3 h. For the GAPDH antibody [GAPDH (14C10) Rabbit mAb #2118, Cell Signaling], a 1:4000 dilution was used.

### Micropatterned PAA hydrogel for TFM experiment

Fibronectin (Corning) was conjugated to acrylic acid N-hydroxysuccinimide (acrylic-NHS) (Sigma-Aldrich) at a concentration of fibronectin (0.1 mg/ml) and acrylic-NHS (4 mg/ml) in 1× PBS for 1 h at room temperature. Following conjugation, the solution was coated on PDMS stamps patterned with arrays of rectangles (17 × 118 *μ*m; 1:7 aspect ratio) and circles (50 *μ*m diameter) for 1 h at room temperature. The stamps were rinsed once with water and air-dried before microcontact printing the conjugated fibronectin onto clean glass coverslips to get the micropatterned coverslips for making polyacrylamide (PAA) gels.

PAA gels of 7.9 kPa stiffness were made by adjusting acrylamide and bisacrylamide stock solution (BioRad Laboratories) concentrations.^41^ A solution of 40% acrylamide, 2% bisacrylamide, and 1× PBS was polymerized by adding tetramethylethylenediamine (Fisher BioReagents) and 1% ammonium persulfate. A droplet of the gel solution supplemented with 0.2 *μ*m fluorescent beads [FluoSpheres™ Carboxylate-Modified Microspheres, 0.2 *μ*m, dark red fluorescent (660/680)] solution was deposited on a glass-bottom dish (MatTek) that was plasma-activated for 1 min and treated with a solution of 2% (v/v) 3-(trimethoxysilyl)propyl methacrylate (Sigma-Aldrich) and 1% acetic acid in ethanol for 10 min. Micropatterned coverslips were placed fibronectin side down on the gel droplet, allowing the gel solution to incorporate the acrylic–NHS–fibronectin complex during polymerization. Gels were polymerized for 45 min at room temperature and soaked in 1× PBS before the coverslips were carefully detached from the gel. Gel substrates were washed with 1× PBS, UV-sterilized, and stored in 1× PBS until cell seeding. Immediately before cell seeding, gels were incubated in a fibronectin solution (0.1 mg/ml) at 37 °C for 30 min.

### Traction force microscopy

TFM measurements were obtained 7 days after cell seeding. Time-lapse images of beads moving near the substrate surface, distributed in and around the contact region of a single cell or cell patch, were acquired at 30 frames/s with a Yokogawa CSU-21/Zeiss Axiovert 200 M inverted spinning disk microscope with a Zeiss LD C-Apochromat 40×, 1.1 NA water-immersion objective and an ORCA-100 camera (Hamamatsu), while the cells were spontaneously beating on the microscope stage equipped with a temperature and CO_2_-equilibrated environmental chamber.

The traction forces exerted by hiPSC-CMs on the gel substrates were computed by measuring the displacement of fluorescent beads embedded within the gel with reference to the beads' positions at peak relaxation, as described previously (software available at https://sites.google.com/site/qingzongtseng/tfm).^42,43^

### FRET imaging and analysis, and sarcomere spacing and order parameter calculation

All FRET imaging was performed on an inverted confocal microscope (Zeiss LSM 710, 63× NA 1.46 objective). For Clover, the excitation wavelength was 488 nm, and the emission range was 500–550 nm. For mRuby2, the excitation wavelength was 561 nm, and the emission range was 600–680 nm. To accurately determine FRET efficiency in contracted and relaxed states, we followed an acceptor (mRuby2, 561 nm, 100 *μ*s pixel dwell time for photobleaching) photobleaching method for determining the FRET efficiency of the ACTN-TS and ACTN-TS-T sensors [supplementary material Fig. 3(a)].^17^ While photobleaching mRuby2, a significant amount of Clover may also get photobleached and as such bias the FRET efficiency calculation. To test this, we incorporated the ACTN-Clov construct into hIPSC cardiomyocytes and photobleached the construct at 561 nm. We observed no significant difference between fluorescence intensities of Clover in CMs before and after 561 nm laser exposure [supplementary material Fig. 4(b)]. This confirms that photobleaching mRuby2 has no or minimal effect on Clover emission. FRET efficiency was calculated using the following equation: E_FRET_ = 1 − F_DA_/F_D_, where F_DA_ is the fluorescence of Donor (Clover) in the presence of Acceptor (mRuby2) and F_D_ is the fluorescence of Donor in the absence of Acceptor (after Acceptor photobleaching). We used a custom MATLAB script to measure the FRET intensities, sarcomere spacing, and order parameter. We first preprocessed the images with morphological top-hat filtering and auto-adjusted the image intensities to saturate the bottom 1% and top 1% of all pixels. We then performed neighborhood thresholding at two length scales to generate binarized masks of the sarcomeres. We determined the total number of sarcomeres from these binary images and located the sarcomeres' centroids. We also used the binary sarcomere masks to compute the average FRET intensity at each sarcomere. To calculate the local sarcomere spacing and order, we iteratively performed the following steps at each sarcomere: We took an image patch (grayscale image) centered around each sarcomere and performed 2D autocorrelation with the image patch. From these 2D autocorrelation maps, we determined the direction of maximum sarcomere periodicity with spacings in the range of 1.2–2.8 *μ*m. We used the direction of local periodicity to extract intensity profiles from which we computed the local sarcomere spacing.

The overall FRET intensities and sarcomere spacings we report are averaged across all sarcomeres in an image. To determine the overall sarcomere orientational order across an image, we calculated the order parameter, $\left\langle \frac{3}{2}{\text{cos}}^{2}\theta -\frac{1}{2}\right\rangle$, where θ is the angle between the direction of local sarcomere alignment at each sarcomere and the director $\hat{n}$, which is the global vector of maximum order, and “⟨ ⟩” is the mean value. Examples of cells with different order parameters are presented in the supplementary material Fig. 5.

### Statistical analysis

All statistical analyses were performed in GraphPad Prism 10. Normality tests indicated non-normal distribution of FRET index values, sarcomere spacing, and order parameter in one or more conditions. Therefore, the Kruskal–Wallis test was conducted to compare multiple groups. A P-value ≤ 0.05 was considered statistically significant.

### Computational simulations

In order to simulate contraction of a cardiomyocyte, a modified version of the chemo-mechanical feedback model of Shenoy *et al.*^31^ was employed. This model describes using thermodynamic principles how cellular contractility and strain change as a function of applied stress taking into account how myosin motor recruitment leads to an overall decrease in free energy within the cell through associated ATP hydrolysis. An adherent, contractile cell generates stresses in its environment and at the cell membrane, which in return can trigger mechanosensitive pathways to increase myosin activation,^32,33,35^ promoting a higher equilibrium density of active myosin motors [Fig. 4(a)]. Without energy input, it is unfavorable for myosin motors to engage the actin cytoskeleton and exert contractile forces due to the chemical energy required for myosin to bind to actin and the strain energy required to deform the cytoskeleton and environment during contraction. However, ATP hydrolysis powers this process and results in an overall reduction in energy. We employ the following formulation for the free energy density, U, to describe actomyosin activity in a three-dimensional setting

$$U=\underset{\text{strain energy}}{\underset{︸}{\frac{K}{2}{\left(\epsilon_{kk}\right)}^{2}+\mu \tilde{\epsilon_{ij}}\tilde{\epsilon_{ij}}}}\underset{\text{external work}}{\underset{︸}{-\frac{1}{3}\int_{0}^{\epsilon_{kk}}\sigma_{kk}d\epsilon_{kk}-\int_{0}^{\tilde{\epsilon_{ij}}}\tilde{\sigma_{ij}}d\tilde{\epsilon_{ij}}}}+\underset{\text{motor binding energy}}{\underset{︸}{\frac{\beta }{6}{\left(\rho_{kk}-3\rho_{0}\right)}^{2}+\frac{\beta }{2}\tilde{\rho_{ij}}\tilde{\rho_{ij}}}}\underset{\text{energy of ATP hydrolysis}}{\underset{︸}{-\frac{1}{3}\int_{0}^{\rho_{kk}}\left(\alpha \sigma_{kk}+\alpha^{a}\sigma_{kk}^{a}\right)d\rho_{kk}-\int_{0}^{\tilde{\rho_{ij}}}(\alpha \tilde{\sigma_{ij}}+\alpha^{a}\tilde{\sigma_{ij}^{a}})d\tilde{\rho_{ij}}}}+\underset{\text{motor work}}{\underset{︸}{\frac{1}{3}\rho_{kk}\epsilon_{kk}+\tilde{\rho_{ij}}\tilde{\epsilon_{ij}}}},$$ (1)

where the stress, strain, and contractility are denoted by the tensors $\sigma_{ij},\;\epsilon_{ij},$ and $\rho_{ij}$, respectively, and in the above definition are separated into their volumetric and deviatoric parts, the latter of which is denoted with a tilde. K and μ represent the bulk and shear moduli of the cytoskeleton, and the parameter $\rho_{0}$ is the value of cell contractility in the quiescent, non-adhered state in the absence of any mechanosignaling. The parameters α, $\alpha^{a}$, and β are chemo-mechanical feedback parameters that represent the strength of mechanosensitive pathways, leading to motor recruitment ( α and $\alpha^{a}$) and the energetic cost of motor binding ( β). More specifically, α reflects the strength of feedback coupling contractility ( $\rho_{ij}$) to stress ( $\sigma_{ij}$), while $\alpha^{a}$ reflects the strength of feedback coupling contractility ( $\rho_{ij}$) to stress anisotropy given by the tensor $\sigma_{ij}^{a}$. This tensor is defined as

$$\sigma_{ij}^{a}=\theta \left(\frac{\sigma_{1}}{\sigma_{2}}\right)\sigma_{1}\;(n_{i}⊗n_{j}),$$ (2)

where $\sigma_{1}>\sigma_{2}>\sigma_{3}$ represent the principal stresses of $\sigma_{ij}$, n is the unit vector representing the direction of the first principal stress, and θ is a unit step function from 0 to 1 centered at 1. The components of $\sigma_{ij}^{a}$ encode whether anisotropy exists within the stress field developed in the contracting cell, as well as the direction in which largest principal stress manifests.

While it can generally be assumed non-muscle actomyosin produces isotropic contractility at the cellular level, sarcomeric actomyosin produces anisotropic contractile forces along the direction of alignment. Following the assumption that sarcomeres align along the direction of first principal stress, we explicitly account for these two types of contraction, both of which are regulated through chemo-mechanical feedback, by including feedback coupled to both $\sigma_{ij}$ and $\sigma_{ij}^{a}$ in Eq. (1). As indicated, the energetic costs considered in Eq. (1) consist of the strain energy of the cytoskeleton, the external work the cell does on the environment as it contracts, the chemical energy required for myosin to bind to actin, and the work done by motors on the cytoskeleton and ECM. Providing the energy to drive contractility in the face of these costs is energy from ATP hydrolysis, which is coupled to feedback from mechanosensitive pathways through stress.

In previous work,^31^ it is shown how steady-state stress–strain relations may be obtained from Eq. (1), as well as the effective moduli of the cytoskeleton components against which myosin contracts. Following the same procedure, we obtain

$$\begin{array}{c} \sigma_{kk}=3\bar{\rho_{v}}+3\bar{K}\epsilon_{kk},\;\tilde{\sigma_{ij}}=2\bar{\rho_{a}}\tilde{\sigma_{ij}^{a}}+2\bar{\mu }\tilde{\epsilon_{ij},}\\ 3\bar{\rho_{v}}=\frac{3\beta \rho_{0}}{\beta -\alpha }+\frac{\alpha^{a}}{\beta -\alpha }\sigma_{kk}^{a},\;3\bar{K}=\frac{3K\beta -1}{\beta -\alpha },\;2\bar{\mu }=\frac{2\mu \beta -1}{\beta -\alpha },\;2\bar{\rho_{a}}=\frac{\alpha^{a}}{\beta -\alpha }. \end{array}$$ (3)

Mechanically, these relations describe a system in which a contractile element (myosin motors) applies compressive stresses to an elastic element representing actin and other passive components of the cell's cytoskeleton. To extend this model to examine the mechanical stress and strain on the individual components of interest within the sarcomere, in particular α-actinin, we additionally include an elastic element in series with these parallel elements that experiences tensile stress upon myosin contraction [Fig. 4(a)]. This arrangement reflects the structure of the sarcomere in which α-actinin bridges the connection between bundles of contractile actomyosin. The stress balance of this system now dictates

$$\sigma_{kk}=3\bar{\rho_{v}}+3\bar{K}\epsilon_{kk}^{\left(1\right)}=3K_{\alpha }\epsilon_{kk}^{\left(2\right)}\;\tilde{\sigma_{ij}}=2\bar{\mu }{\tilde{\epsilon_{ij}}}^{\left(1\right)}+2\bar{\rho_{a}}\tilde{\sigma_{ij}^{a}}=2\mu_{\alpha }{\tilde{\epsilon_{ij}}}^{\left(2\right)},$$ (4)

where the moduli of α-actinin are represented by $K_{\alpha }$ and $\mu_{\alpha }$, and the strains on actomyosin and α-actinin are represented by $\epsilon_{ij}^{\left(1\right)}$ and $\epsilon_{ij}^{\left(2\right)}$, respectively. Solving this system to find the total stress ( $\sigma_{ij}^{\text{total}}$) in terms of total strain [ $\epsilon_{kk}^{\text{total}}=\epsilon_{kk}^{\left(1\right)}+\epsilon_{kk}^{\left(2\right)}$] leads to

$$\begin{array}{c} \sigma_{kk}^{\text{total}}={3K}_{\text{eff}}\epsilon_{kk}^{\text{total}}+3\rho_{\text{eff}}^{v},\;\tilde{\sigma_{ij}^{\text{total}}}=2\mu_{\text{eff}}\tilde{\epsilon_{ij}^{\text{total}}}+2\rho_{\text{eff}}^{a}\tilde{\sigma_{ij}^{a}},\\ 3K_{\text{eff}}=\frac{3K_{\alpha }\bar{K}}{K_{\alpha }+\bar{K}},\;3\rho_{\text{eff}}^{v}=\frac{3K_{\alpha }}{K_{\alpha }+\bar{K}}\bar{\rho_{v}},\;2\mu_{\text{eff}}=\frac{2\mu_{\alpha }\bar{\mu }}{\bar{\mu }+\mu_{\alpha }},\;{2\rho }_{\text{eff}}^{a}=\frac{2\bar{\rho_{a}}\mu_{a}}{\bar{\mu }+\mu_{a}}. \end{array}$$ (5)

Equation (5) describes the steady-state relations for stress and strain in the sarcomere. We utilize finite element analysis in COMSOL Multiphysics^®^ (Version 6.3) to implement and solve these relations for circular and rectangular cell geometries and obtain the predicted stress and strain at every point in the cell. The rectangular cell geometry is modeled as a capped half-cylinder 120 *μ*m in length and 12.5 *μ*m in radius. The circular cell was modeled with a half-sphere of equivalent volume. These cell geometries were assigned the effective bulk and shear moduli given by Eq. (4) and placed on a linear elastic block of substrate with Young's modulus 7.9 kPa and Poisson's ratio 0.45 to represent the polyacrylamide substrate on which experimental TFM measurements were performed. The edges and bottom of the block were fixed, and cell contractility was implemented as an external stress tensor of value $\rho_{\text{eff}}^{v}\delta_{ij}+2\rho_{\text{eff}}^{a}\tilde{\sigma_{ij}^{a}\;}$ as given by Eq. (5). Values used for other model parameters are listed in Table I, and any parameters not listed were assigned the same values as used by Shenoy *et al.*^31^ The moduli of α-actinin, $K_{\alpha }$ and $\mu_{\alpha }$, were approximated with the same values as the cytoskeleton bulk and shear moduli. To model cells in the relaxed as opposed to the contracted state, a very small value of $\rho_{0}=10$ Pa was used.

The order parameter in our simulations was determined by calculating the invariant tensor $Q_{ij}$ as commonly done when studying molecular orientation such as in the case of a nematic liquid crystal.^44^ We assume stress fibers and sarcomeres develop along the directions of principal stresses in the cell body as predicted in our simulations, and therefore utilize the unit vector describing the direction of the first principal stress, n, to provide a prediction of sarcomere orientation at every point in the cell. The tensor $Q_{ij}$ is then calculated at every point according to the following formula:

$$Q_{ij}=\frac{1}{2}\left(3n_{i}n_{j}-\delta_{ij}\right).$$ (6)

In the case of real molecules, one may perform a summation of this expression over all molecules to construct the tensor $Q_{ij}$, but in the case of our coarse-grain simulation, we calculate $Q_{ij}$ at every point within the cell body and then take a volume average. The largest eigenvalue of the resulting average $Q_{ij}$ is reported as the order parameter.

## SUPPLEMENTARY MATERIAL

See the supplementary material for the validation of the α-actinin-2 tension sensor, visual references of the sarcomere order parameter, and the primers used for the generation of the tension sensor.

## Data Availability

The data that support the findings of this study are available from the corresponding authors upon request.
